# Cardiovascular risk and mortality in end-stage renal disease patients undergoing dialysis: sleep study, pulmonary function, respiratory mechanics, upper airway collapsibility, autonomic nervous activity, depression, anxiety, stress and quality of life: a prospective, double blind, randomized controlled clinical trial

**DOI:** 10.1186/1471-2369-14-215

**Published:** 2013-10-08

**Authors:** Israel dos Reis Santos, Aline Roberta Danaga, Isabella de Carvalho Aguiar, Ezequiel Fernandes Oliveira, Ismael Souza Dias, Jessica Julioti Urbano, Aline Almeida Martins, Leonardo Macario Ferraz, Nina Teixeira Fonsêca, Virgilio Fernandes, Vinicius Alves Thomaz Fernandes, Viviane Cristina Delgado Lopes, Fernando Sérgio Studart Leitão Filho, Sérgio Roberto Nacif, Paulo de Tarso Camillo de Carvalho, Luciana Maria Malosá Sampaio, Lílian Christiane Giannasi, Salvatore Romano, Giuseppe Insalaco, Ana Karina Fachini Araujo, Humberto Dellê, Nadia Karina Guimarães Souza, Daniel Giannella-Neto, Luis Vicente Franco Oliveira

**Affiliations:** 1 Rehabilitation Sciences Master Degree and PhD Program, Nove de Julho University (UNINOVE), São Paulo, SP, Brazil; 2 Physical Therapy School, Nove de Julho University (UNINOVE), São Paulo, SP, Brazil; 3 Psychology School, Health Department, Nove de Julho University (UNINOVE), São Paulo, SP, Brazil; 4 Physical Therapy School, Universidade Estadual de Ciências da Saúde de Alagoas (UNCISAL), Maceió, AL, Brazil; 5 Centro de Nefrologia Zona Norte (CENENORTE), São Paulo, SP, Brazil; 6 Medicine School, Fortaleza University (UNIFOR), Fortaleza, CE, Brazil; 7 Bioscience Department, Universidade Estadual Paulista (UNESP/SJC), São Paulo, SP, Brazil; 8 Istituto di Biomedicina e di Immunologia Molecolare “Alberto Monroy” (IBIM), CNR, Palermo, SI, Italy; 9 Medicine Master Degree Program, Nove de Julho University (UNINOVE), São Paulo, SP, Brazil; 10 Sleep Laboratory, Nove de Julho University (UNINOVE), São Paulo, SP, Brazil

**Keywords:** Chronic kidney disease, Dialysis, Sleep, Quality of life

## Abstract

**Background:**

Chronic kidney disease (CKD) is one of the most serious public health problems. The increasing prevalence of CKD in developed and developing countries has led to a global epidemic. The hypothesis proposed is that patients undergoing dialysis would experience a marked negative influence on physiological variables of sleep and autonomic nervous system activity, compromising quality of life.

**Methods/Design:**

A prospective, consecutive, double blind, randomized controlled clinical trial is proposed to address the effect of dialysis on sleep, pulmonary function, respiratory mechanics, upper airway collapsibility, autonomic nervous activity, depression, anxiety, stress and quality of life in patients with CKD. The measurement protocol will include body weight (kg); height (cm); body mass index calculated as weight/height^2^; circumferences (cm) of the neck, waist, and hip; heart and respiratory rates; blood pressures; Mallampati index; tonsil index; heart rate variability; maximum ventilatory pressures; negative expiratory pressure test, and polysomnography (sleep study), as well as the administration of specific questionnaires addressing sleep apnea, excessive daytime sleepiness, depression, anxiety, stress, and quality of life.

**Discussion:**

CKD is a major public health problem worldwide, and its incidence has increased in part by the increased life expectancy and increasing number of cases of diabetes mellitus and hypertension. Sleep disorders are common in patients with renal insufficiency. Our hypothesis is that the weather weight gain due to volume overload observed during interdialytic period will influence the degree of collapsibility of the upper airway due to narrowing and predispose to upper airway occlusion during sleep, and to investigate the negative influences of haemodialysis in the physiological variables of sleep, and autonomic nervous system, and respiratory mechanics and thereby compromise the quality of life of patients.

**Trial registration:**

The protocol for this study is registered with the Brazilian Registry of Clinical Trials (ReBEC RBR-7yhr4w and World Health Organization under Universal Trial Number UTN: U1111-1127-9390 [http://www.ensaiosclinicos.gov.br/rg/RBR-7yhr4w/]).

## Background

Chronic kidney disease (CKD) is one of the most serious public health problems. The increasing prevalence of this disease in developed and developing countries has led to a global epidemic. Current epidemiological surveys suggest that approximately 1 million individuals with terminal CKD have undergone kidney replacement therapy worldwide. A large part of this epidemic may be explained by the increase in the number of diabetes mellitus cases and the increase in life expectancy. Diabetic nephropathy is expected to affect approximately 5.4% of the world population by 2015 [1].

CKD is defined as the presence of kidney damage or reduced kidney function for ≥3 months, regardless of the diagnosis. The advanced stage of this condition is known as terminal CKD or end-stage kidney disease, with progressive and irreversible loss of kidney function [2].

CKD is classified on the basis of the glomerular filtration rate (GFR), as recommended by the US National Kidney Foundation Kidney Disease Outcomes Quality Initiative, which provides the basis for the management of this disease. CKD is classified into 5 stages: stage 1, kidney damage with normal or increased GFR ≥ 90 mL/(min·1.73 m^2^); stage 2, kidney damage with mildly decreased GFR of 60 to 89 mL/(min·1.73 m^2^); stage 3, moderately decreased GFR of 30 to 59 mL/(min·1.73 m^2^); stage 4, severely decreased GFR of 15 to 29 mL/(min·1.73 m^2^); and stage 5, kidney failure with a GFR < 15 mL/(min·1.73 m^2^). Evidence of kidney damage for ≥3 months is required for the diagnosis of stage 1 and 2 CKD, as manifested by pathological kidney abnormalities or abnormal urine composition (such as haematuria or proteinuria) or abnormalities on imaging tests [3].

The prevalence rate of sleep disorders in patients with CKD ranges from 40% to 80%, which is higher than those in the general population [4]. The most frequent comorbidities include diabetes mellitus type 2 [1], periodic leg movements during sleep [4], obstructive sleep apnea (OSA) and nocturnal hypoxemia [5], dyslipidaemia, coronary disease, heart failure [6,7], systemic arterial hypertension [8,9], respiratory disorders [10,11], stress[12 ], depression [12-14], anxiety [15].

OSA is a respiratory disease characterised by the collapse of the upper airways during sleep in predisposed subjects. After chronic obstructive pulmonary disease and asthma, OSA is the most important and widespread respiratory disease, affecting 3% to 7% of the male population and 2% to 5% of the female population between 40 and 65 years of age in the Western world [16]. In Brazil, the prevalence of OSA is even higher at 24.8% in men and 9.6% in women, according to a recent epidemiological study conducted in the city of São Paulo [17].

The causal association between OSA and CKD or whether the 2 diseases result from a common pathophysiologic process has not yet been clarified. Uremic milieu has been implicated as a cause of OSA in patients with CKD. Altered ventilatory drive and chemoreceptors can lead to decreased respiration via a blunted response to ventilatory stimuli, such as hypoxia or acidaemia. Upper airway obstruction can occur from localised oedema or the collapse of the dilator muscles, leading to an increased risk of OSA. Suppression of the respiratory musculature due to metabolic acidaemia/acidosis, osmotic disequilibrium, and a reduction in middle molecule clearance could potentially cause or contribute to OSA [18].

### Aims and hypotheses

The present study involving patients with CKD was designed with 4 main objectives: (1) to assess the effects of dialysis on sleep parameters and determine the prevalence and severity of sleep disorders; (2) to determine the behaviour of sympathetic and parasympathetic autonomic nervous system activity through an analysis of heart rate variability; (3) to detect upper airway collapsibility as a risk indicator for OSA; and (4) to evaluate levels of depression, anxiety, stress and quality of life. Our hypothesis is that the weather weight gain due to volume overload observed during interdialytic period will influence the degree of collapsibility of the upper airway due to narrowing and predispose to upper airway occlusion during sleep, and to investigate the effects of nocturnal hemodialysis compared to daytime hemodialysis and the influences in the physiological variables of sleep, autonomic nervous system (ANS), and respiratory mechanics and thereby compromise the quality of life in chronic kidney diseases and end-stage renal disease patients undergoing dialysis.

## Methods/Design

### Study design

**Figure 1:**
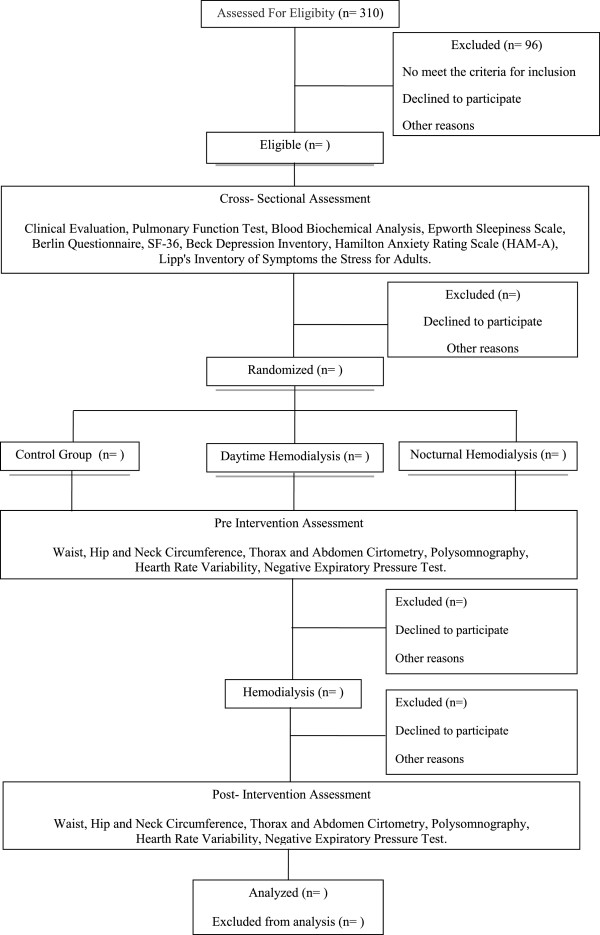
Flowchart of the study protocol.

A prospective, consecutive, double-blind, randomized controlled clinical trial is proposed to investigate the effect of dialysis on sleep, pulmonary function, respiratory mechanics, upper airway collapsibility, ANS, depression, anxiety, stress and quality of life in end-stage renal disease patients, as summarised in Figure 1.

### Subjects

The participants will be recruited from the Centro de Nefrologia Zona Norte (São Paulo, Brazil) and sent to the Sleep Laboratory of the Universidade Nove de Julho (São Paulo, Brazil). Patients will be recruited consecutively and screened for eligibility using a standardised protocol. The following will be used as the inclusion criteria: male or female patients aged 18 to 80 years; chronic kidney failure; candidate for kidney transplant with indication for dialysis; cognitive level sufficient for understanding the procedures and following the instructions; and agreement to participate by signing a statement of informed consent. Meanwhile, the following will used as the exclusion criteria: craniofacial abnormalities; undergoing active treatment of sleep apnea; active malignancy; active alcohol and/or drug abuse; and dementia or treatment-refractory psychiatric diseases leading to an inability to provide informed consent.

### Randomization

After the initial assessment and compliance with eligibility criteria, participants will be randomized into two intervention group [patients achieving diurnal hemodialysis and nocturnal hemodialysis] and the control group [CKD not submitted to dialysis]. Randomization numbers will be generated using a randomization table at a central office. A series of numbered, sealed, opaque envelopes will be used to ensure confidentiality. Each envelope will contain a card stipulating to which group the subject will be allocated.All patients underwent randomization protocol that will meet the eligibility criteria and be clinically stable.

### Blinding

Baseline data will be collected prior to randomisation so that trial investigators, assessors, research co-ordinators and trial participants will be blinded to allocation while the data is being collected. Blinding will be maintained in all steps of the research.

### Sample size calculation and statistical analysis

According to the findings of a previously published epidemiological study [17] that the proportion of adults with an apnea/hypopnoea index (AHI) ≥ 15 corresponds to 16.9% of the Brazilian population and that of a previous study [18] that AHI ≥ 15 had an approximately 50% prevalence rate in patients with CKD undergoing conventional dialysis (3 times a week, 4 h per session) [19], the sample size was calculated as 57 patients, with an *α* = 0.05 and *β* = 90% (test power).

The Kolmogorov–Smirnov normality test will first be performed to determine whether a normal distribution sample is present. A descriptive analysis will be performed, with the data expressed as either mean and standard deviation or median values and 95% confidence intervals, as appropriate. One-way analysis of variance will be used for comparisons between work shifts once the samples have a normal distribution. V_0.2_ and ΔV (%) values will be linearly correlated with the AHI, for which either the Pearson or Spearman correlation test will be used, depending on the sample distribution. Either the non-paired Student *t* test or Mann–Whitney test will be used for comparisons between individuals with and those without OSA.

In addition, a logistic regression analysis of continuous factors with categorical responses will be performed. Receiver operating characteristic (ROC) curves will be constructed to determine the sensitivity (true-positive rate) versus 100% specificity (false-positive rate) at various levels of the measured $\Delta \dot{V}$ (%) and V_0.2_ (%) to identify the cut-off value yielding the largest number of correctly classified patients. The statistical analysis will be performed by an experienced statistician using the JMP version 8.0 (SAS/STAT® Software, SAS Institute Inc., Cary, NC, USA) and SPSS version 16.0 programs (Somers NY). A 5% level of significance and 95% confidence interval will be applied.

### Ethical considerations

The study will be conducted in accordance with the ethical standards established in the 1961 Declaration of Helsinki (as revised in Hong Kong in 1989 and Edinburgh, Scotland, in 2000) and is in compliance with the Regulatory Guidelines and Norms for Research Involving Human Subjects of the National Health Board of the Brazilian Health Ministry issued in October 1996. This protocol received approval from the Human Research Ethics Committee of Universidade Nove de Julho (Brazil) under process no. 368856/2010 and is registered with the World Health Organization under Universal Trial Number (UTN) U1111-1127-9390 and the Brazilian Registry of Clinical Trials (REBEC no. RBR-7YHR4W). All participants will be required to sign a statement of informed consent and will be allowed to withdraw from the study at any time with no negative consequences. All the procedures of the study will be confidential.

### Evaluation protocol

#### Clinical evaluation

Patients with chronic kidney diseases and end-stage renal disease undergoing dialysis, based on recommended procedures, will be submitted to general physical measurements performed by a well-trained physician and physical therapist before and after the dialysis session using precise instruments. The measurement protocol will include body weight (kg); height (cm); body mass index (BMI) calculated using the formula weight/height^2^[16]; circumferences (cm) of the neck, waist, and hip [20]; heart and respiratory rates; blood pressures; Mallampati index [21]; tonsil index [22]; heart rate variability [23]; maximum ventilatory pressure; negative expiratory pressure (NEP) test; and polysomnography (PSG; sleep study), as well as the administration of specific questionnaires addressing sleep apnea, excessive daytime sleepiness [24,25], symptoms of stress [12], and depression [12-14], anxiety [15], and quality of life [26,27].

The following data will be obtained from medical charts: biochemical values, associated pathological conditions, laboratory values (iron, intact-parathyroid hormone, haemoglobin, calcium, phosphorus, and creatinine), duration of dialysis therapy, comorbidities, and aetiology of kidney disease.

#### Physical examination

Weight and height determinations will be performed using an electronic scale (model 200/5; Welmy Industria e Comercio Ltda, São Paulo, Brazil), and the BMI will be calculated [16]. For the assessment of the tonsils and Mallampati index scoring, each subject will be in the sitting position and instructed to open the mouth as wide as possible [21,22].

#### Waist and neck circumferences

Measurements of waist and neck circumferences will be performed with a metric tape (7 mm in width). The sites for the measurements will be standardised. Waist circumference will be measured at the midpoint between the lower edge of the last rib and the iliac crest. Neck circumference will be measured horizontally over the cricoid cartilage [28].

#### Thorax and abdomen cirtometry

Cervical-thoracic abdominal cirtometry will be performed to assess thorax and abdomen mobility and define the diaphragm index. The measurement will be performed by fixing the zero point on the metric tape to the anterior region of the thorax at the level that is being measured (axillary, xiphoid, or abdominal), with the tape encircling the entire circumference of the thorax or abdomen with maximal possible pressure and the other extremity of the tape placed over the same fixed point. The aim of applying the maximal possible pressure of the tape on the body is to avoid the interference of soft structures in the measurements. Mobility and range of motion will be provided by maximal inspiration and expiration [29].

### Lung function tests

#### Spirometry

Lung function tests will be performed during the day, with the patient seated in a comfortable position. For such, the KoKo PFT Spirometer System version 4.11 (nSpire Health, Inc, Louisville, CO, USA) will be used in accordance with the guidelines for the execution of lung function tests established by the Brazilian Society of Pneumology [30] and European Respiratory Society [31]. The subjects will perform the test in a comfortable position, with the body erect and upper limbs unsupported. All the examinations will be performed by a competent technician trained in obtaining the necessary cooperation from the subjects and appropriately operating the equipment to ensure accurate, reproducible results. The spirometer will be calibrated before each exam using a 3-L syringe [30].

#### Analysis of respiratory mechanics

Maximal inspiratory pressure and maximal expiratory pressure physiologically constitute the most adequate test for the determination of ventilatory muscle strength. Maximal inspiratory pressure is an indicator of ventilatory capacity and the development of respiratory failure, the measurement of which is indicated for the assessment of the degree of abnormality and monitoring of the weakening of individual inspiratory muscles in disease progression [32]. The tests will be performed on the same day on which the patients will undergo spirometry. The tests will be performed in a quiet setting. The patients will be seated comfortably, breathing calmly and at rest, with the trunk at a 90-degree angle in relation to the thighs [30].

### Sleep evaluation

#### Berlin questionnaire

The Berlin questionnaire is used to identify patients at high risk of respiratory sleep disorders in a variety of populations. This clinical history questionnaire has recognised efficacy in distinguishing individuals at high risk of OSA, with 10 items organised into 3 categories as follows: snoring and apnea (5 items), daytime sleepiness (4 items), and systemic arterial hypertension and obesity (1 item). All marked responses are considered positive. The score is divided into the following categories: category 1 is considered positive in the occurrence of 2 or more positive responses to items 1 to 5; category 2, in the occurrence of 2 or more positive responses to items 6 to 8; and category 3, when the response to item 9 is “yes” or when the BMI ≥ 30 kg/m^2^. Two or more positive categories indicate a high risk of OSA [24].

#### Epworth Sleepiness Scale

The Epworth Sleepiness Scale is a simple, self-administered questionnaire with items addressing situations involving activities of daily living and the occurrence of daytime sleepiness in adults. The subjects will be instructed to classify their likelihood of feeling the desire to nap or sleep in 8 situations on a scale of 0 to 3 (0, no chance of napping; 1, small chance of napping; 2, moderate chance of napping; and 3, strong chance of napping) [25,33].

#### Polysomnography

All the patients will undergo a standard overnight PSG (Embla, A10 version 3.1.2 Flaga, Hs. Medical Devices, Reykjavik, Iceland) at the Sleep Laboratory of Nove de Julho University*.* Polysomnography exams will be held the night before the haemodialysis and approximately 12 hours after this dialysis. All recording sensors will be attached to the patient in a non-invasive manner using tape or elastic bands. The following physiological variables will be monitored simultaneously and continuously: 4 channels for the electroencephalogram (C3-A2, C4-A1, O1-A2, and O2-A1), 2 channels for the electrooculogram (EOG-Left-A2 and EOG-Right-A1), 4 channels for the surface electromyogram (muscles of the submentonian region, anterior tibialis muscle, masseter region, and seventh intercostal space), electrocardiogram (derivation V1 modified), airflow detection via 2 channels through a thermocouple and nasal pressure cannula, respiratory effort of the thorax and abdomen via x-trace belts, snoring and body position sensors, and arterial oxygen saturation and pulse rate via an oximeter.

All the subjects will be monitored by a technician experienced in PSG. Sleep stages will be visually scored in 30-s epochs, and each PSG recording will be analysed manually under blinded conditions by the same examiner with experience in scoring PSG recordings in accordance with international standards established by the Academy of Sleep Medicine Manual for Scoring Sleep and Associated Events. The patients will be instructed to remain as relaxed as possible and sleep naturally, as if at home. All signals will be recorded continuously [34,35].

#### NEP test

The NEP test will be conducted before and after hemodialysis sessions, performed through the administration of a negative pressure at the mouth during expiration. This is a practical test performed while awake and requires little cooperation from the subject. In the absence of expiratory flow limitation, the increase in the 8 pressure gradients between the alveoli and the open upper airway caused by NEP results in an increase in expiratory flow.

**Figure 2:**
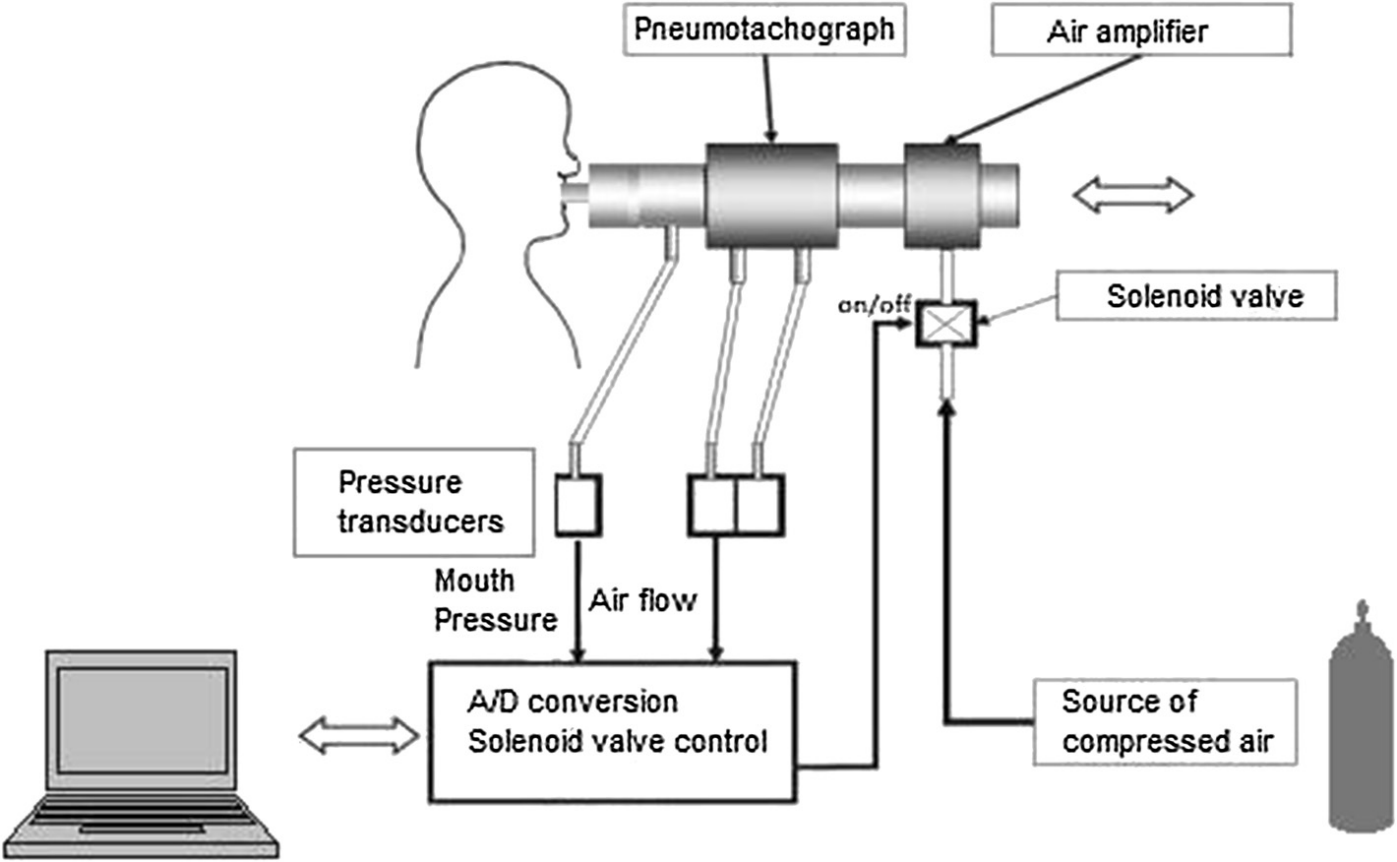
Schematic representation of the negative expiratory pressure.

NEP will be generated by the Super Air Amplifier (Exair model 120021, Cincinnati, OH, USA) coupled to a tank of compressed air via an electrically operated solenoid valve (model 95004, Norgren Ltd; Vimercate, MI, Italy) automatically activated in early expiration and kept open for 2 s by software control (Figure 2). A pneumotachograph (Hans Rudolph model 3830; Kansas, MO, USA) will be connected to the air amplifier and the mouthpiece to measure airflow ($\dot{V}$) with pressure transducers (PCLA02X5; Sensortechnics GmbH, Puchheim, Germany). Mouth pressure will be measured by pressure transducers (PCLA0050; Sensortechnics GmbH, Puchheim, Germany). NEP of 10-cm H_2_O will be set by occluding the pneumotachograph with a stopper and adjusting the flow of compressed air-to-air amplifier (Figure 2).

Each NEP manoeuvre will be performed each after at least 4 breaths to normalise the breathing pattern. The tests will be performed once with the subject seated comfortably and again in supine position on a cot. In both positions, care will be taken to maintain the comfort of the subjects, with the neck in a neutral position, as it has been documented that the position of the head exerts an influence on upper airway collapsibility [36].

All manoeuvres will be performed with the subjects awake and wearing a nose clip. The airflow and mouth pressure signals will be low-pass filtered and sampled at 200 Hz. Both digital signals will be displayed in real time on the monitor and stored on the computer for subsequent analysis. Signal analysis and solenoid valve control will be performed using software written in 9 LabVIEW 8.2 (National Instruments) developed by the Italian National Research Council, Institute of Biomedicine and Molecular Immunology “A. Monroy.”

NEP application during tidal expiration produces an immediate peak flow followed by a sudden drop of a variable degree. Upper airway collapsibility is to be evaluated by measuring flow limitation as flow drop ($\Delta \dot{V}$) expressed as the percentage of peak flow immediately after NEP administration. To avoid reflex and voluntary reactions to the NEP stimulus, the minimum flow will be identified in the first 200 ms of NEP administration [37].

Upper airway collapsibility will also evaluated by measuring V0.2 immediately after NEP administration. These values will be expressed as the percentage of mean inspiratory volume of the 3 breaths preceding NEP administration. Measured volumes will be accepted only when differences between inspiration and expiration for each of the 3 previous breaths are less than 10%. Values of V_0.2_ and $\Delta \dot{V}$ (%) are calculated as the mean of 4 measurements. Measurements of upper airway collapsibility will be evaluated as expiratory volume in 0.2 s (percentage of the mean inspiratory volume of the 3 breaths preceding NEP application) and as flow drop ($\Delta \dot{V}$), expressed as the percentage of peak flow.

### Assessment of ANS

Nerve-Express is a fully automatic, non-invasive computer-based system designed for the quantitative assessment of the ANS on the basis of the analysis of heart rate variability. This system is based on a method of clusterisation of the relationship between sympathetic and parasympathetic nervous system statuses.

This technological breakthrough is achieved by using proprietary algorithms and a new approach based on a leading theory of artificial intelligence, the Marvin Minsky’s Frame Theory. Nerve-Express provides an objective and reliable real-time evaluation of the ANS state during the orthostatic test and Valsalva manoeuvre combined with deep breathing.

A signal sensor is attached to the thorax by an elastic strap and is coupled to the Nerve-Express software program, which collects and stores the data. The modality of the exam is the orthostatic test, in which the patient goes from the supine to the orthostatic position [38,39].

### Inventory of symptoms of stress for adults

The Inventory of Symptoms of Stress for Adults (LIPP) is based on a 4-phase model of stress and its manifestations in the somatic and cognitive domains. In the initial stress phase, which is called the alert phase, the body makes greater efforts to prepare itself to cope with a stressor. This phase is considered a positive phase of stress because it is important for adjustments to environmental demands.

The second phase is called resistance and occurs when a chronic stressor demands coping for a long period of time. This phase is associated with fatigue, perception of burnout, and cognitive loss. The next phases, referred to as almost exhaustion and exhaustion, are consequences of the breakdown of resistance and loss of capacity for adjustment. During these phases, one can observe important changes in sleep, work, and libido in addition to symptoms of anxiety and depression [12].

### Hamilton anxiety rating scale (HAM-A)

The term depression is used to describe a clinical syndrome, where there are several particular signs and symptoms. Depression can occur as a primary manifestation of mood, associated with systemic medical illnesses, other psychiatric disorders or disorders arising from the use of psychoactive substances. With regard to the study of depression, has not, to date, the biological or physiological parameters to evaluate its clinical manifestations in a definitive or conclusive. So the depression scales emerge as useful tools for assessing the severity of depressive, serving to translate clinical phenomenon in objective and quantitative information. In addition to characterizing the intensity of depression, these scales would serve to evaluate the response to treatment when applied before, during and after therapeutic intervention.

Within this context, the rating scales of depression severity, whose first date from the end of the last century can be divided into ranges: a) self-assessment, b) hetero c) mixed, involving self and observer.[15]

The first scale of hetero-evaluation, that is, applied by an observer, was the Hamilton Rating Scale for Depression (HAM-D), designed and developed by Hamilton in the late 50s. Currently, it is the most widely used depression scale worldwide and is probably the most important being regarded as “standard gold” in assessing the severity of depression compared with new and used rating scales in order to check the reliability of these.

Rating Scale Hamilton Anxiety is one of the most atualemnte used to assess anxiety in patients and is composed of 14 items (6 items - Humor anxious and 8 items - Physical symptoms) that pertain to the observed behavior. The patient will respond from their own experience the classification of symptoms present in Likert scale where 0 = absent, 1 = mild, 2 = medium intensity, 3 = 4 = strong intensity and moderate intensity.

The total score reflects the general state of the patient’s anxiety, the aspects considered by the scale for the assessment are: mood, cognition, behavior, alertness and somatic symptoms, and in addition to other symptoms, scores between 0 and 17 are considered normal, between 18:25 26 moderate and 30 severe, over 30 are uncommon, but show a severe state of anxiety [40,41].

### Beck depression inventory

The Beck Depression Inventory (BDI) has 21 items, including attitude, depressive symptoms, and suicidal ideation, scored on a scale ranging from 0 to 3. The cut-off scores are as follows: <11, minimal depression; 12 to 19, mild to moderate depression; 20 to 35, moderate depression; and 36 to 63, severe depression [12].

### Quality of life

Patients with CKD may develop eating disorders due to the diet and water intake restrictions during treatment, which may also result in dysfunctional behaviour and treatment noncompliance. Thus, stress, anxiety, and depression are common among such patients and imply a reduction in quality of life. The Short Form 36 (SF-36) will be used for the assessment of quality of life. This measure has 36 questions grouped into 8 domains or scales of physical functioning, role limitations due to physical health, bodily pain, general health perception, social functioning, and role limitations due to emotional problems, vitality, and mental health [26,27].

### Study interventions

#### Haemodialysis

Regular daytime or nocturnal haemodialysis was standardised during the trial. It was performed 3 times per week, with a 4-h session duration, 250-mL/min blood flow, and 500-mL/min dialysate flow, using bicarbonate-buffered dialysate with 1.25 mmol/L ionised calcium concentration, dialysate temperature of 36.5°C, and Polyflux 17-L dialyser [42].

The ultrafiltration amount for each haemodialysis session was decided by individual dry weight, which was fixed during the trial. In addition, during the trial, the patients were not permitted to change their medication or start new drugs, especially antiplatelet agents, angiotensin-converting enzyme inhibitors, angiotensin II receptor antagonists, calcium-channel blockers, and β-blockers.

The patients who required a change in medication for medical reasons were subsequently excluded from the study. Use of *N*-acetylcysteine was also prohibited in view of its potential influence on ischemia-reperfusion injury.

#### Quality control

To ensure the quality of the data, the physiotherapists and physicians in charge of data collection will receive specific training. Periodic external monitoring will be performed to verify the adequate application of methodology in performing examinations and data collection.

## Discussion

CKD is a major public health problem worldwide, and its incidence has increased in part by the increased life expectancy and increasing number of cases of diabetes mellitus and hypertension. Sleep disorders are common in patients with renal insufficiency and is much more prevalent in patients with end-stage renal disease than in the general population [4].

Symptomatic obstructive sleep apnoea syndrome has been related to be a risk factor for hypertension, heart failure and vascular dysfunction, and has been proposed to be causally related to both non-fatal and fatal coronary and cerebrovascular events [43]. Moreover, renal chronic patients frequently presented restless legs syndrome that also is associated with higher mortality and elevated incidence of cardiovascular events [44]. Thus, once presence of OSA also seems to reduce quality of haemodialysis [45] it’s incorrectly identification among CKD patients may lead to worst outcomes or fail of hypertension control [46].

Our hypothesis is that the weather weight gain due to volume overload observed during interdialytic period will influence the degree of collapsibility of the upper airway due to narrowing and predispose to upper airway occlusion during sleep, and to investigate the influences of different shifts haemodialysis in the physiological variables of sleep and ANS activity, and respiratory mechanics, and depression, anxiety, stress and quality of life in end-stage renal disease patients. With our proposed study, we intend to identify the main factors that cause sleep disorders in patients with CKD that lead to the identification of the best treatment to reduce mortality and improve the quality of life of these patients.

## Competing interests

The authors declare that they have no competing interests.

## Authors’ contributions

LVFO, GI, DGN, ARD, AKFA, and IRS provided the concept of the study, created the hypothesis, and wrote the original proposal. IRS, ICA, FSSLF, ARD, DGN, and LVFO significantly contributed to the writing of this proposal. ISD, EFO, VCDL, JJU, NTF, LMF, AAM, and IRS participated in the data collection, VF, VATF, and SRN performed clinical evaluations, FSSLF, PTCC, LCG, SR, GI, and LVFO were involved in the critical revision of the manuscript. This protocol paper was written by IRS, DGN, ARD, GI and LVFO, with input from all co-authors. All the authors read and approved the final manuscript.

## Pre-publication history

The pre-publication history for this paper can be accessed here:

http://www.biomedcentral.com/1471-2369/14/215/prepub
